# Smoking prevalence, determinants, knowledge, attitudes and habits among Buddhist monks in Lao PDR

**DOI:** 10.1186/1756-0500-2-100

**Published:** 2009-06-08

**Authors:** Sychareun Vanphanom, Alongkon Phengsavanh, Visanou Hansana, Sing Menorath, Tanja Tomson

**Affiliations:** 1 Postgraduate Studies & Research Department, University of Health Sciences, Lao PDR, PO Box 7444, Vientiane, Lao PDR; 2 Dept. of Public Health Sciences, Division of Social Medicine, Karolinska Institutet, SE-171 77 Stockholm, Sweden

## Abstract

**Background:**

This cross-sectional study, the first of its kind, uses baseline data on smoking prevalence among Buddhist monks in Northern and Central provinces of Lao PDR.

**Findings:**

Between March and September 2006, 390 monks were interviewed, using questionnaires, to assess smoking prevalence including determinants, knowledge and attitudes. Data entry was performed with Epi-Info (version 6.04) and data analysis with SPSS version 11. Descriptive analysis was employed for all independent and dependent variables. Chi-square or Fisher's exact test were used for categorical variables to compare smoking status, knowledge, attitudes and province. Logistic regression was applied to identify determinants of smoking. Daily current smoking was 11.8%. Controlling for confounding variables, age at start of monkhood and the length of religious education were significant determinants of smoking. The majority of the monks 67.9% were in favor of the idea that offerings of cigarettes should be prohibited and that they should refuse the cigarettes offered to them (30.3%) but, in fact, 34.8% of the monks who were current smokers accepted cigarettes from the public.

**Conclusion:**

Some monks were smokers, whilst they, in fact, should be used as non-smoking role models. There was no anti-smoking policy in temples. This needs to be addressed when setting up smoke-free policies at temples.

## Background

Every six seconds, someone dies of a smoking-related disease [1]. By 2030, more than 80% of tobacco-related deaths will be in low- and middle-income countries [2]. The tobacco epidemic is one of the greatest public health challenges not least in the Western Pacific and South East Asia.

The Lao People's Democratic Republic (Lao PDR) is one of the poorest countries in the world with a Gross National Income (GNI) per capita of $ 935 or less, life expectancy at birth of 63 years and under-five mortality rate of 75 [3,4]. Tobacco is listed as the third most important agricultural crop in Lao PDR and this is, obviously, in conflict with any tobacco control policy [5]. Daily smoking among males is 50% and females 10% [2] and this is the first study on smoking among Buddhist monks in Lao PDR.

More than 85% of the population in Lao PDR are Buddhist[4], shaping the country's religious, ethnic, and cultural identity [6]. Monks are the main religious practitioners and most young men are expected to become a monk for a short period of their lives. In many societies in South Asia, the act of offering a cigarette is described as an important "exchange". Surprisingly, as monks are supposed to be detached from this kind of pleasure, tobacco is offered to monks in a ceremony or 'Sukhouan'.

In Laos, Buddhism provides guidelines for behavior through its five precepts for the laity: refrain from taking life, from stealing, from illicit sexual activity, from speaking falsely, and from consuming inebriating substances. However, cigarettes are not included as illicit drugs and there is no existing policy of prohibition of smoking among monks. The monks are not only in charge of Buddhist religious ceremonies but function as dream interpreters, traditional medical practitioners, and counselors. Thus, monks function as role models and, given their central role in Lao culture [7] there may be a potential for successful cooperation with monks in tobacco control efforts.

The aim of this study was to assess smoking prevalence, including its determinants, among Buddhist monks/novices and their knowledge of, and attitudes towards smoking.

## Methods

This was a cross-sectional, quantitative study identifying baseline data on smoking prevalence among monks in the Northern and Central provinces of the Lao PDR, Louang Prabang and Vientiane. Most temples are in the Central and Northern parts of the country. Data collection was done between March and September 2006.

In 2006, there were 4111 temples with 11,582 monks and 12,463 novices. The total numbers of monks and novices in Vientiane and Luangprabang was 6,180. The selection of districts was based on the concentration of temples, i.e. purposive sampling for the provinces and districts. In each selected district, the list of monks/novices who were to take part in the study in each of the temples [8] was achieved through systematic, random selection of a set proportion of the number of monks/novices in the temple. The inclusion criteria for monks were: having been a monk at least 1 year; age 12 to 35 years and being able to answer a face-to-face-administered questionnaire.

The sample size was calculated based on the simple population formula [9]. Due to lack of information on the proportion of smoking among monks, the calculation was based on the prevalence of smoking among males in Lao PDR (41%) [10]. Assuming a confidence level of 95 percent at the 5 percent level of significance and a precision of 5 percent, the total sample size was 390 monks and novices (300 monks in Vientiane Capital City and 90 monks in Luangprabang using proportionate random sampling size).

The validated questionnaire was based on the World Health Organization Global tobacco survey among health professionals [11] adapted to the current target group. Variables assessed were: age, sex, smoking history (duration, frequency, and previous attempts to quit), socio-economic status (education), and other smoking variables (presence or absence of other smokers in family and temple, whether friends and fellow monks smoked), knowledge, attitudes and beliefs regarding smoking. The smoking data was categorized as never smoker (those who had never tried a cigarette in their lifetime), former/ex-smoker (those who ever smoked, but had stopped now) and current smokers (those who reported smoking during the study both occasionally and daily). Data was collected by medical doctors from the University of Health Sciences, Vientiane, Lao PDR.

Knowledge was assessed dichotomously (1 = yes and 0 = no). Questions on knowledge of the effects of smoking on health were summed, with a high score indicating high knowledge and a low score denoting low knowledge. Attitudes towards smoking were assessed from answers with an ordinal Likert scale which ranged from 1 (strongly disagree), 2 (disagree), 3 (agree), to 4 (strongly agree). Similarly scores on attitudes were totaled, we summed the strongly disagree and disagree to be negative attitudes and agree and strongly agree to be positive attitudes. The highest score of attitudes was 3.30, meaning positive attitudes and lower scores indicating negative attitudes towards smoking.

### Analysis

Data entry was performed with Epi-Info (version 6.04), and analysis with SPSS version 11. Descriptive analysis was employed for all independent and dependent variables. Chi-square or Fisher's exact tests (Chi2 when normal and Fischer's when high percentages) for categorical variables were used to compare smoking status, knowledge, attitudes and provinces. Logistic regression was applied to identify determinants of smoking while controlling for confounding variables such as socio-demographics, history of monkhood, family member smoking, public offerings of cigarettes, knowledge, attitudes and beliefs regarding smoking.

Ethical clearance was obtained from the Relevant Ethical Review Board at the Faculty of Medical Sciences, Ministry of Education with Ref: 138/NECHR, dated 25 February 2006.

## Results

### Socio-demographic characteristics of the study population

Three hundred and ninety monks/novices participated in this study (Table 1) with a mean age of 19.84 ± 5.47 (SD). About 87.2% of the monks and novices received religious education and 39.4% had 1 to 2 year religious education with a mean of religious education of 3.58 years. The mean age of initiation to monkhood was 14.78 ± 4.41 (SD) and 65.4% of respondents had been monks/novices up to 5 years, reflecting the status of fully-pledged monks or novices.

**Table 1:** Characteristics of monks and novices

Variables	Total	
	
	N	%
Age (Mean = 19.84; Min = 12; Max = 45; SD = 5.47)		
≤ 14 yrs	36	9.2
15 – 24 yrs	296	75.9
≥ 25 yrs	58	14.9
Religious Education		
Yes	340	87.2
No	50	12.8
If yes, how many years (Mean = 3.58; SD = 2.410)		
1 – 2 yrs	134	39.4
3 – 4 yrs	107	31.5
5 – 6 yrs	69	20.3
7 – 15 yrs	30	8.8
Age initiation of monkhood (Mean = 14.78; Min = 8; Max = 45; SD = 4.41)		
≤ 14 yrs	228	58.5
15 – 24 yrs	152	39.0
≥ 25 yrs	10	2.5
Duration of monkhood (Mean = 4.92; Min = 0; Max = 28; SD = 4.07)		
≤ 5 yrs	255	65.4
6 – 10 yrs	94	24.1
>11 yrs	41	10.5
Status of monks/novices		
Monks, fully-pledged	148	37.9
Novices	242	62.1
Administrative position		
Abbot/Administrative	20	5.1
Monk/Novice	370	94.9
Family member smoking		
No	111	28.5
Yes	279	71.5
Public offered cigarettes to monks		
No	336	86.2
Yes	54	13.8

### Smoking habits of monks and novices

About 11.8% of the monks and novices surveyed were current daily smokers, 10.3% occasional smokers, 27.2% former smokers and 50.7% non-smokers. Among 106 former smokers, 21.7% had smoked daily. About 51.9% started smoking before monkhood.

### Current Smokers

The majority started smoking on a regular basis at the age 15–20 years (72.1%) with a mean age 17.2 (range 10–30) (Table 2). It is interesting to note that 79.1% reported that they started smoking during monkhood. One quarter (25.6%) reported that they had smoked for 1 year; 24.4% had smoked from 3 to 4 years and 19.8% had smoked more than 10 years.

**Table 2:** Current status of smoking behavior among monks and novices

	Total	
	
Variables	N	%
Age of first trying cigarette on regular basis (Mean = 17.23; SD = 3.238; Min = 10; Max = 30)		
≤ 14 yrs	13	15.1
15 – 20 yrs	62	72.1
≥ 21 yrs	11	12.8
When did you start smoking		
Before becoming monk/novice	18	20.9
During period of monkhood	68	79.1
How long did you smoke regularly (Mean = 5.10; SD = 5.14; Min = 1; Max = 21)		
1 y	22	25.6
2 yrs	14	16.3
3 – 4 yrs	21	24.4
5–9 yrs	12	13.9
≥ 10 yrs	17	19.8
N° of cigarettes smoked during a day (Mean = 7.64; SD = 8.83; Min = 1; Max = 40)		
1–3 cs	34	39.5
4 – 9 cs	26	30.2
10 – 19 cs	15	17.5
≥ 20 cs	11	12.8
Have you smoked 100 (or more) cigarettes in your life		
Yes	71	82.6
No	15	17.4
First cigarette: How long after getting up/waking up		
≤ 15 mns	29	33.7
15 – 30 mns	7	8.1
≤ 1 hr	36	41.9
Others	14	16.3
Reason for starting smoking (Multiple responses)		
Boredom	4	3.4
Peer pressure	58	48.7
To relieve stress	21	17.7
To imitate adults	11	9.2
Did not buy/received for free	8	6.7
Reduce hunger	3	2.5
Others	14	11.8
Getting cigarettes from:		
Buy	62	45
Get from People	34	24.6
Get from fellow monk	33	23.9
Others	9	6.5

Most smoked 1 to 3 cigarettes per day (39.5%); followed by 4 to 9 cigarettes per day (30.2%). About one-third of the respondents (33.7%) reported having their first cigarette less than 15 minutes after waking up and 41.9% had their first cigarette less than 1 hour after awakening.

The reasons stated for starting smoking were: peer influence (48.7%), stress relief (17.6%), imitation of adults (9.2%) and obtaining cigarettes for free (6.7%). When asked how they got cigarettes, 44.9% bought their own cigarettes, 24.6% received cigarettes offered by the general public and 23.9% got cigarettes from their fellow monks.

### Quitting smoking

About 97.7% of monks and novices want to quit smoking. Among those who desired to quit, the main reasons for smoking cessation were the following: 40.7% wanted to avoid illnesses; 22.1% mentioned having illness at or before quitting and 12.8% saw illnesses occurring in some smokers. Not knowing how to quit was the main reason given for not being able to quit smoking (Table 3).

**Table 3:** Percentage distribution of smoking cessation among monks and novices

	Total	
	
Variables	N	%
Want to quit smoking		
Yes	84	97.7
No	2	2.3
Tried to quit smoking within last year		
Yes	66	76.7
No	20	23.3
How long did you stop smoking		
≤ 1 month	31	47
1 – 5 months	28	42.4
6 – 11 months	4	6.1
1 year	2	3
2 years or longer	1	1.5
Methods used to quit smoking		
'Cold turkey'	8	18.2
Drug therapy	2	4.5
Weaning	34	77.3
Others	22	50
Received advice to quit smoking		
Yes	43	65.2
No	23	34.8
Person giving advice to quit smoking (Multiple response)		
Doctor/Nurse	7	53.8
Lay people	13	22.4
Fellow monks	21	36.2
Media	4	6.9
Others	20	34.5
Primary reason for quitting smoking		
Illness (at or before time of quitting),	19	22.1
Healthy, but wanted to prevent illness	35	40.7
Seeing Illness develop in other smokers	11	12.8
Family Disapproval	5	5.8
Not enough money to buy tobacco	2	2.3
Disapproval of friends and co-workers	2	2.3
Don't know/refuse to answer	12	14.0
Reason not to quit smoking		
Don't know how	31	36

Just don't want to	5	5.8
No advice	5	5.8
Others	45	52.3

About three-quarters (76.7%) of the respondents reported that they had ever tried to quit smoking within the last year. Around half 47% had stopped smoking for less than 1 month, 42.4% (had) stopped smoking for 1 to 5 months. The majority (77.3%) used the weaning method (to progressively wean smokers from the smoking habit) and 18.2% used the 'cold turkey method' (expression describing the actions of a person who gives up a habit or addiction all at once) for quitting. Most frequently, doctors and nurses had advised them to quit (53.8%); followed by fellow monks (36.2%).

### Knowledge of the health effects of smoking and attitudes towards smoking

Respondents in all groups had favorable levels of knowledge in almost all statements except for the items: *Tobacco kills more people each year than illegal drugs, AIDS, and car crashes; knows about smoking laws in religious places; and knows their temple's smoking rule *(Table 4).

**Table 4:** Monk's and novice's knowledge and attitudes by smoking status

Variables	Non smoker		Current smoker		Total		*p-value
	**Correct**		**Correct**		**Correct**		

**Knowledge of smoking on health**	**N**	**%**	**N**	**%**	**N**	**%**	

a. Smoking is harmful	303	99.7	85	98.8	388	99.5	.393
b. Nicotine in tobacco is highly addictive	266	87.5	73	84.9	339	86.9	.587
c. People can get addicted to cigarettes like they can get addicted to cocaine or heroin**	256	84.2	60	69.8	316	81.0	.005
d. Passive smoking increases the risk of heart disease in non-smoking adults	261	85.9	71	82.6	332	85.1	.492
e. Passive smoking increases the risk of lung diseases in non-smoking adults	293	96.4	82	95.3	375	96.2	.750
f. Smoking increases the risk of heart diseases	264	86.8	73	84.9	337	86.4	.721
g. Smoking increases the risk of LRI	296	97.4	82	95.3	378	96.9	.308
h. Tobacco kills more people each year than illegal drugs, AIDS & car crashes	160	52.6	41	47.7	201	51.5	.464
i. Quitting smoking reduces risk	295	97.0	84	97.7	379	97.2	1.000
j. Know about smoking law in religious places*	145	47.7	53	61.6	198	50.8	.028
k. Know their temple's smoking rule	127	41.8	44	51.2	171	43.8	.140
l. Smoke from cigarettes is harmful to people who are repeated exposed	290	95.4	81	94.2	371	95.1	.582

**Attitudes towards anti-smoking activities**	Positive		Positive		Positive		*p-value

	N	%	N	%	N	%	
a. Smoking in all enclosed public places be banned	276	90.8	79	91.9	355	91	1.000
b. Smoking should be banned at the temple	264	86.6	67	77.9	331	84.9	.059
c. Offering tobacco to the monks should be prohibited*	215	70.7	50	58.1	265	67.9	.036
d. Monks should refuse cigarettes offered to them	99	32.6	19	22.1	118	30.3	.064
e. If Monks don't smoke, people would respect more	276	90.8	74	86	350	89.7	.227
f. Monks should routinely advise people to quit smoking*	288	94.7	75	87.2	363	93.1	.027
g. There should be campaign to the public not to offer cigarettes to monks	250	82.2	64	74.4	314	80.5	.123
h. There should be a project to quit smoking or smoking cessation for smoking monks/novices	289	95.1	80	93.0	369	94.6	.427
i. People did not accept monks who smoke (smoking monks)	196	64.5	56	65.1	252	64.6	1.000
j. Monks who use tobacco are less likely to advise people stop smoking	222	73.0	60	69.8	282	72.3	.586

*P-value from Chi2-test and Fischer's exact test

Non-smokers had better knowledge, and represented the largest group (84.2%) who knew that people can get addicted to tobacco in a similar manner as one can become addicted to narcotics. The comparative figure for smokers is 69.8% (p < .005). Less than half of the monks knew about the smoking rules in the temples, smokers knowing more often than non-smokers (61.6% versus 47.7%; P < .05 respectively).

Regarding attitudes towards smoking, most agreed that smoking should be banned in all enclosed public places and that smoking should be banned at temples. Two-thirds (67.9%) of all the monks agreed that offering tobacco to monks should be prohibited. Non-smokers agreed that monks should refuse the cigarettes offered to them more often than smokers (70.7% versus 58.1%, P < .05). Non-smokers held more positive attitudes towards the statement *"Monks should routinely advise people to quit smoking" *than smokers (94.7% versus 87.2%, P < .05).

### Factors related to smoking

Smoking behavior of monks and novices was positively correlated with younger age, lower educational level, late age at monkhood, being a monk/novice, less duration of monkhood and less years of religious education (Table 5). After controlling for confounders, age at start of monkhood and more years of religious education were significant determinants of smoking. Individuals starting monkhood at late age and having more years of religious education had a higher odds of smoking (OR = 1.84 and OR = 4.92 respectively) compared to those who started monkhood at early age and those who had less years of education (p = .034 & p = .004 respectively), (Table 5).

**Table 5:** Logistic Regression of smoking status among monks and novices

Predictors^a^	Smokers		OR	95%CI
	**N**	**%**		

Age				

12 – 24 years old	63	73.3	1	

25 – 45 years old	23	26.7	1.72	.73–4.02

Age at start of monkhood				

≤ 14 years old	40	46.5	1	

≥ 14 years old	46	53.5	1.84	1.04 – 3.26

Administrative position				

Abbot/Administrative	9	10.5	1	

Monk/Novice	77	89.5	.68	.20 – 2.34

Family member smoking				

No	28	32.6	1	

Yes	58	67.4	.65	.35 – 1.22

Public offered cigarettes to monks				

No	72	83.7	1	

Yes	14	16.3	1.09	.49 – 2.43

Duration of religious education				

1–5 years	46	66.7	1	

6–8 years	15	21.7	2.00	.97–4.12

≥ 8 years	8	11.6	4.92	1.43–16.94

Knowledge of health effect of smoking				

Poor*	36	41.9	1	

Good**	50	58.1	.99	.56 – 1.75

Attitudes toward smoking				

Negative^+^	37	43.0	1	

Positive^++^	49	57.0	.84	.47 – 1.48

• *Poor knowledge ≤ 79% of total score of knowledge

• **Good knowledge ≥ 80% of total score of knowledge

• ^+^Negative attitude ≤ 79% of total score of attitudes

• ^++^Positive attitudes ≥ 80% of total score of attitudes

## Discussion

This is the first study on smoking among Buddhist monks in Lao PDR. The overall prevalence of daily current smoking among monks in the two provinces was 11.8%, lower than in Thailand (24.4%) [12] in Cambodia (44%) [8] and in the general population in Lao PDR (41%) [10]. The results probably reflect the prevalence in the general population in each country. Even if the prevalence in Lao PDR was low, the monks and novices in our study have a high level of dependency on tobacco with about 42% having their first cigarette less than one hour after awakening.

The findings suggest that monks have a high knowledge of the harmful effects of smoking on health, as did monks in Cambodia [8]. Clearly, being offered cigarettes free of charge increases the risks of being addicted. In fact, 24.6% of the monks who are current smokers received cigarettes from the public. An individual's friends' influence was responsible for almost half of all reported reasons for starting smoking.

Sixty-eight percent of the respondents stated that offering cigarettes should be prohibited, and one-third stated that they should refuse the cigarettes offered to them. The fact that as many as two-thirds of the monks consider receiving cigarettes could be explained by the fact that the general population consider this to be merit-worthy behavior. The act of offering cigarettes as alms to their ancestors is seen as important, and, if monks refuse these alms, ancestors do not receive the merits offered by relatives. Additionally, according to Lao custom and tradition, it is impolite and disrespectful for monks to refuse things offered to them.

In a Cambodian study it was found that about one-third (34%) of all respondents thought that people should not offer cigarettes to monks [8]. In theory, a majority of monks were against smoking, including accepting cigarettes as gifts, but, in practice, one-third accepted this kind of offering.

Our study revealed that the majority of monks (74.9%) said that there was no smoke-free policy in place at temples. This is in contrast to the tobacco-free Ministry of Health. However, some abbots have their own standards on smoking in their temples, for example prohibiting smoking on premises such as within temple buildings, and in front of the public. These findings are in accordance with our data which showed that there were no official regulations and laws related to smoking. Almost all of the Cambodian monks (91%) were clear that the teachings of Buddha said nothing about smoking regulations and seventy-one percent of them recommended a law for Buddhist monks not to smoke [8]. A Thai study among Buddhist monks also found that smoking might be unwise, but did not have a moral dimension [13]. Most Buddhists do not consider that smoking violates the five precepts. However, the fact that age at start of monkhood and years of religious education were associated with monks' smoking [12,13]. The latter could be explained by the fact that monks with longer religious education actually learn more about the five precepts including drug addiction and tobacco use.

As in all studies of this nature, recall bias may occur. In addition, due to self-esteem, some monks may have under- or over-reported their smoking status. The interviewers tried to establish trust and to ensure confidentiality and privacy to keep this bias to a minimum. The assessment of current smoking status was not validated by biomarkers such as nicotine and exhaled carbon monoxide [14]. The questionnaire was adapted from the validated World Health Organization Global Tobacco survey among health professionals [11].

Overall, the data highlights the fact monks in Lao PDR are also smokers, that there is no anti-smoking policy in temples, and there is ambiguity regarding the offering of cigarettes. Monks should be used as "role models' not to smoke as the majority of Lao people believe in Buddhism and the role of Buddhism in daily life is crucial.

## Policy Implications

To capitalize on Buddhist monks as role models there is a need for a nationwide comprehensive approach including smoke-free regulations at all temples. Cigarettes should be seen as the unhealthy addictive dependency product they are and must be firmly detached from the spiritual development and ideals that Buddhism represents. Also, monks should get assistance in smoking cessation and obviously, in addition, training to become counselors regarding this particular addiction which is so detrimental for public health, not least in resource-poor settings.

## Competing interests

The authors declare that they have no competing interests.

## Authors' contributions

VS, the project initiator, made substantial contributions to conception and design, acquisition, analysis and interpretation of data. AP, VH, contributed in supervision of data collection, analyzing data and writing the first draft. TT, made substantial contributions in analyzing and interpretation and was involved in drafting the manuscript and revising it critically for important intellectual content. All authors have given final approval of the version to be published. Each author participated sufficiently in the work to take public responsibility for appropriate portions of the content.
